# *Wolbachia w*Pip Blocks Zika Virus Transovarial Transmission in *Aedes albopictus*

**DOI:** 10.1128/spectrum.02633-21

**Published:** 2022-07-27

**Authors:** Yan Guo, Jiatian Guo, Yifeng Li

**Affiliations:** a Guangdong Provincial Key Laboratory of High Technology for Plant Protection, Plant Protection Research Institute, Guangdong Academy of Agricultural Science, Guangzhou, Guangdong, China; b Key Laboratory of Tropical Disease Control of the Ministry of Education, Zhongshan School of Medicine, Sun Yat-Sen University, Guangzhou, Guangdong, China; University of Mississippi

**Keywords:** *Wolbachia*, Zika virus, mosquito, transovarial transmission

## Abstract

*Wolbachia* is being developed as a biological tool to suppress mosquito populations and/or interfere with their transmitted viruses. Adult males with an artificial *Wolbachia* infection have been released, successfully yielding population suppression in multiple field trials. The main characteristic of the artificial *Wolbachia*-infected mosquitoes used in the suppression program is the lower vector competence than that in native infected/uninfected mosquitoes in horizontal and vertical transmission. Our previous studies have demonstrated that the Aedes albopictus HC line infected with a trio of *Wolbachia* strains exhibited almost complete blockade of dengue virus (DENV) and Zika virus (ZIKV) in horizontal and vertical transmission. However, the extent to which *Wolbachia* inhibits virus transovarial transmission is unknown since no studies have been performed to determine whether *Wolbachia* protects ovarian cells against viral infection. Here, we employed ovarian cells of the *Ae*. *albopictus* GUA (a wild-type mosquito line superinfected with two native *Wolbachia* strains, *w*AlbA and *w*AlbB), HC, and GT lines (tetracycline-cured, *Wolbachia*-uninfected mosquitoes), which exhibit key traits, and compared them to better understand how *Wolbachia* inhibits ZIKV transovarial transmission. Our results showed that the infection rate of adult GT progeny was significantly higher than that of GUA progeny during the first and second gonotrophic cycles. In contrast, the infection rates of adult GT and GUA progeny were not significantly different during the third gonotrophic cycle. All examined adult HC progeny from three gonotrophic cycles were negative for ZIKV infection. A strong negative linear correlation existed between *Wolbachia* density and ZIKV load in the ovaries of mosquitoes. Although there is no obvious coexistence area in the ovaries for *Wolbachia* and ZIKV, host immune responses may play a role in *Wolbachia* blocking ZIKV expansion and maintenance in the ovaries of *Ae. albopictus*. These results will aid in understanding *Wolbachia*-ZIKV interactions in mosquitoes.

**IMPORTANCE** Area-wide application of *Wolbachia* to suppress mosquito populations and their transmitted viruses has achieved success in multiple countries. However, the mass release of *Wolbachia*-infected male mosquitoes involves a potential risk of accidentally releasing fertile females. In this study, we employed ovarian cells of the *Ae. albopictus* GUA, HC, and GT lines, which exhibit key traits, and compared them to better understand how *Wolbachia* inhibits ZIKV transovarial transmission. Our results showed an almost complete blockade of ZIKV transmission in HC female mosquitoes. *Wolbachia* in natively infected GUA mosquitoes negative affected ZIKV, and this interference was shown by slightly lower loads than those in HC mosquitoes. Overall, our work helps show how *Wolbachia* blocks ZIKV expansion and maintenance in the ovaries of *Ae. albopictus* and aids in understanding *Wolbachia*-ZIKV interactions in mosquitoes.

## INTRODUCTION

Aedes albopictus (*Ae*. *albopictus*) is an important vector of arboviruses, including Zika (ZIKV) and dengue (DENV), and it is native to eastern and southern Asia but has recently spread worldwide (1). ZIKV is mainly transmitted to humans by *Ae. albopictus*, was introduced into Brazil in 2013 (2), and is now endemic throughout various countries in Asia, Africa, Oceania, and the Americas, causing devastating microcephaly and other neurological disorders (3). In the absence of effective vaccines or antiviral therapies, ZIKV has expanded its geographic range and poses a substantial threat to global public health.

ZIKV is mainly maintained by horizontal transmission between *Aedes* spp. mosquitoes and animals. ZIKV also spreads by vertical transmission, although such transmission in the arthropod vector population is generally considered a maintenance mechanism during conditions which are adverse for horizontal transmission, and it is relatively inefficient (4–6). Vertical transmission in mosquitoes is a maternal phenomenon mostly accomplished by trans-egg and transovarial transmission. In trans-egg transmission, the virus infects eggs during oviposition. In transovarial transmission, the virus enters developing oocytes in the germinal tissues (7, 8). Several members of the *Orthobunyavirus* genus, such as California encephalitis virus in *Aedes dorsalis* (9) and San Angelo virus in *Ae. albopictus* (10), have exhibited the capability for transovarial transmission. According to these studies, transovarial transmission is based on viral infection of the oocyte-forming process, which leads to nearly 100% infection in successive generations (11).

In addition to virus-specific efforts, vector control is the primary intervention tool to stop the spread of arbovirus diseases. The rapid spread of mosquitoes combined with their vector competence for diverse arboviruses is particularly challenging to control using traditional approaches (12). Insecticides are efficient chemicals to reduce populations of larvae and adults, but they have high health and environmental costs (13–15). Sterile insect strategies (SIT) prevent mosquitoes from producing viable offspring by releasing artificially reared radiation-sterilized males into the field to mate with wild females, and they have shown recent success in effectively suppressing Aedes aegypti and *Ae. albopictus* (16, 17). Recent novel vector control strategies have focused on *Wolbachia*-based mosquito population suppression and replacement, including the incompatible insect technique (IIT), in which male mosquitoes infected with *Wolbachia* are released to induce incompatible mating with target females. *Wolbachia* are obligate intracellular bacteria that infect >65% of insect species and many other arthropods (18), and they are maternally transmitted from mother to offspring. In addition, some *Wolbachia* strains interfere with arbovirus replication in mosquitoes, suggesting that this strategy could be widely effective. *Wolbachia*-mediated pathogen inhibition is broadly effective, showing activity against ZIKV, DENV, West Nile virus (WNV), chikungunya virus (CHIKV), yellow fever virus (YFV), and eukaryotic parasites (19–21).

In nature, *Wolbachia*-infected mosquitoes have been demonstrated to have higher viral resistance, although to a lower degree than mosquito-transinfected strains (22–24). *Ae. albopictus* mosquitoes in the field are superinfected with two native *Wolbachia* strains, *w*AlbA and *w*AlbB. The native *Wolbachia* from *Ae*. *albopictus* has been related to lower DENV transmission; however, in a La Réunion population of Ae. albopictus, there was no significant impact on CHIKV transmission (22, 25). Compared to native infected mosquitoes, *Wolbachia*-transinfected mosquitoes are highly related with a viral inhibition phenotype, and the strength of viral inhibition is considered to depend on the density of *Wolbachia* (26, 27). Nevertheless, the extent to which different *Wolbachia* strains inhibit virus transmission has yet to be determined. Such investigations are vital to precisely evaluating the potential role of *Wolbachia* in mosquito virus transmission.

Our laboratory generated the *Ae. albopictus* HC line, which possesses a triple *Wolbachia* infection, by transferring *w*Pip from its native mosquito host Culex pipiens into the *Ae. albopictus* HOU line by embryonic microinjection (17, 28). Our previous studies have demonstrated that *Wolbachia* significantly reduced the vector competence of HC females for both horizontal and vertical transmission of DENV and ZIKV (17). However, the extent to which *Wolbachia* inhibits ZIKV transovarial transmission in *Ae. albopictus* is unknown since no studies have been performed to determine whether *Wolbachia* protects ovarian cells against viral infection. In this study, we examined ovarian cells of the *Ae. albopictus* GUA (a wild-type mosquito strain superinfected with two native *Wolbachia* strains, *w*AlbA and *w*AlbB), HC, and GT lines (tetracycline-cured, *Wolbachia*-uninfected mosquitoes), which exhibit key traits, and compared them to better understand how ZIKV transovarial transmission is inhibited by *Wolbachia* in the GUA and HC lines. These results will aid in understanding *Wolbachia*-ZIKV interactions in mosquitoes.

## RESULTS

### ZIKV vertical transmission.

**FIG 1:**
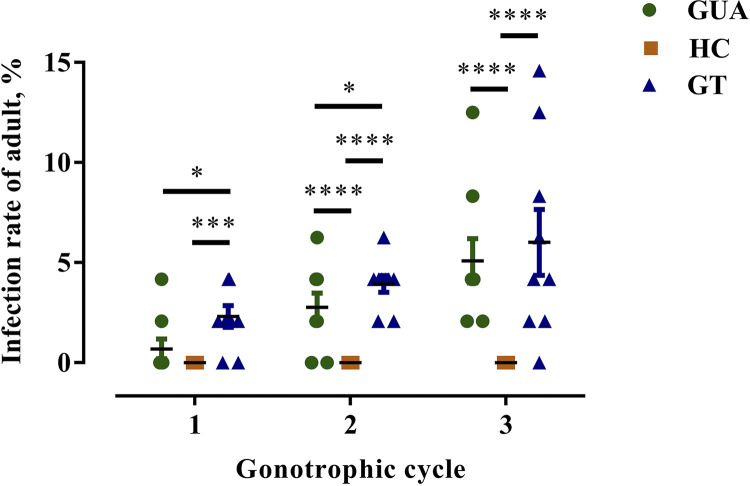
Zika virus (ZIKV) infection in the adults of Aedes albopictus progeny. GUA, HC and GT line mosquitoes were orally infected with ZIKV and allowed to finish the first gonotrophic cycle. After this, mosquitoes of each line were re-fed with pure sheep blood to finish the second and third gonotrophic cycles. Forty-eight adults from each gonotrophic cycle of the inoculated female progeny were randomly selected to test for ZIKV infection. Bars show the infection rates of adult GUA, GT, and GUA progeny ± standard deviations (SDs) (*, *P* < 0.05; ***, *P* < 0.001; ****, *P* < 0.0001).

A total of 120 female GUA, HC, and GT line mosquitoes, fully engorged on freshly propagated ZIKV supernatant (final virus titer: 5.5 × 10^5^ PFU/mL) mixed with sheep blood, were used to evaluate their capacity to transmit ZIKV to progeny. The results showed that the infection rate of the GT progeny was significantly higher than that of the GUA progeny during the first (*P* = 0.0118, two-way analysis of variance [ANOVA]) and second (*P* = 0.0498) gonotrophic cycles. In contrast, the GT and GUA progeny infection rates were not significantly different during the third gonotrophic cycle (*P* = 0.0764) (Fig. 1). All HC progeny samples were negative, with infection rates significantly lower than those of the GUA and GT progeny during the three gonotrophic cycles (Fig. 1). These results suggest that there may be a direct relationship between *Wolbachia* and vertical transmission of ZIKV.

A total of 24 female mosquitos of the GUA, HC, and GT lines that had finished three gonotrophic cycles were dissected (ovaries/legs/carcasses), and PCR was used to check their ZIKV infection status. The results showed that the infection rates of the legs were 83.33%, 79.16%, and 95.83% in GUA, HC, and GT females, respectively. The infection rates of the carcasses were 87.50%, 87.50%, and 95.83% in GUA, HC, and GT females, respectively. All ovarian samples of the HC line were negative (Fig. S1 in Supplemental File 1). The *Wolbachia* titers and ZIKV genome copies of whole bodies were significantly different between mosquitoes and their parents which were infected by blood-feeding (Fig. S2).

### *Wolbachia* distribution in ovaries.

**FIG 2:**
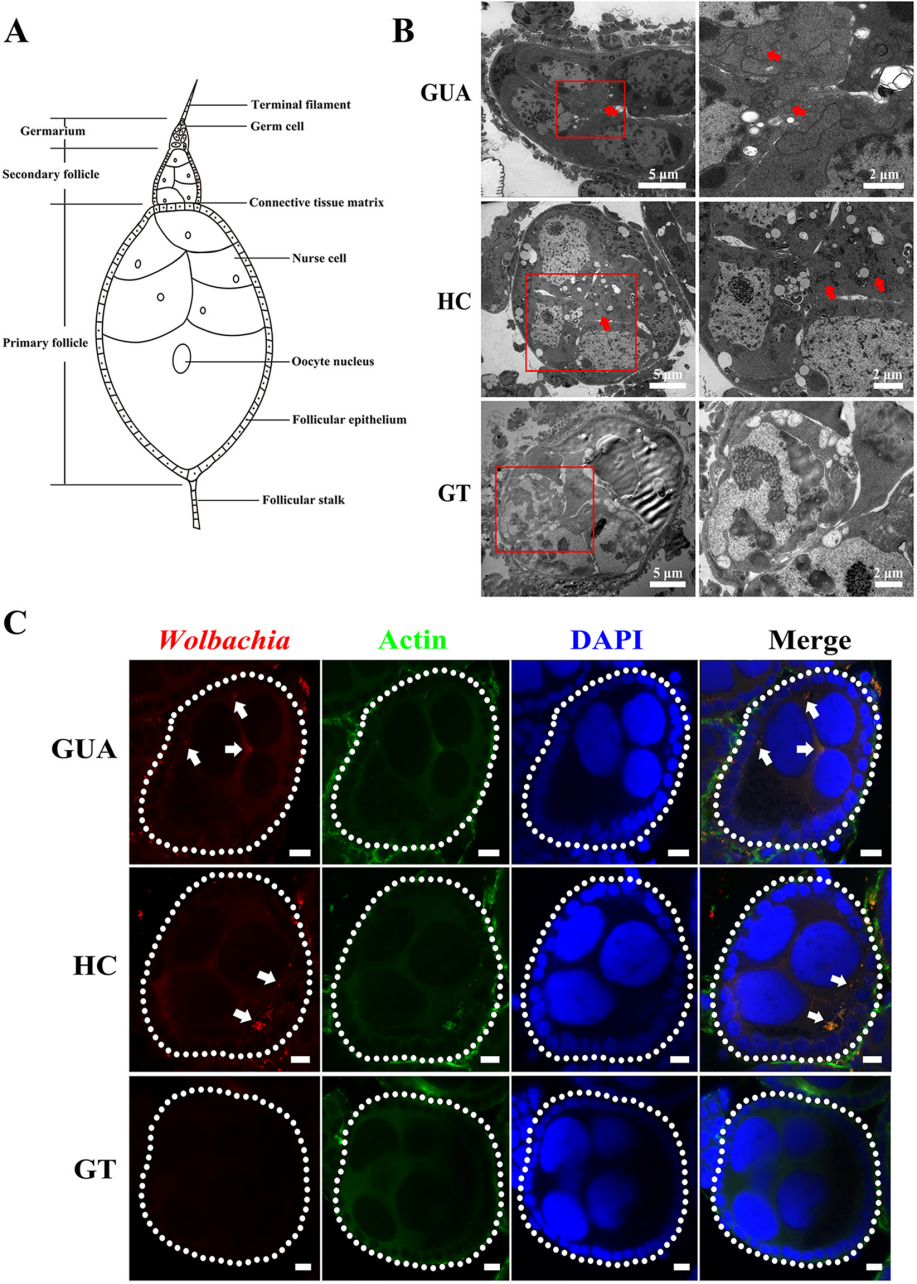
*Wolbachia* distribution in *Ae. albopictus* ovaries. (A) The *Ae. albopictus* ovariole is composed of a terminal filament, germarium, and vitellarium. The germarium connects with the terminal filament, which is composed of germ cells. Below the germarium is the vitellarium. The vitellarium houses 3 to 4 linearly arranged developing follicles, in which oogenesis is completed. (B) Visualization of *Wolbachia* in developing follicles by transmission electron microscopy. Red arrowheads indicate *Wolbachia*. (C) Visualization of *Wolbachia* in developing follicles by immunofluorescence staining. *Wolbachia* were distributed among all ovarian cells, including follicular cells, the extracellular space between nurse cells, and the ooecium of oocytes. White arrowheads indicate *Wolbachia*. Red: *Wolbachia*; green: actin; blue: *Ae. albopictus* DNA. Scale bars: 20 μm.

*Ae. albopictus* ovarioles are of the polytrophic meroistic type, consisting of a terminal filament, germarium, and vitellarium (Fig. 2A). The vitellarium houses 3 to 4 linearly arranged developing follicles which develop from stages I to V (29). Female mosquitoes are ready for oviposition once oogenesis reaches stage V. Egg production is a cyclic process. Therefore, with each successive reproductive or gonotrophic cycle, a batch of oocytes matures and a new set of follicles forms within the germarium, separates, and starts developing (29).

To identify a possible functional role of *Wolbachia* in blocking *Ae. albopictus* transovarial transmission of ZIKV, we tested for its presence in the ovaries and ovarioles using transmission electron microscopy (TEM) and confocal laser scanning microscopy. *Wolbachia* signals were found inside the germarium and vitellarium, dispersed throughout the follicles in the GUA and HC mosquitoes (Fig. S3). In follicles, *Wolbachia* were present in follicular cells, the extracellular space between nurse cells, and the ooecium of oocytes (Fig. 2B and C, arrowheads). No *Wolbachia* signals were found in the ovaries or ovarioles of the GT line (Fig. 2B and C, Fig. S3).

### *Wolbachia* inhibit ZIKV entry into ovaries.

**FIG 3:**
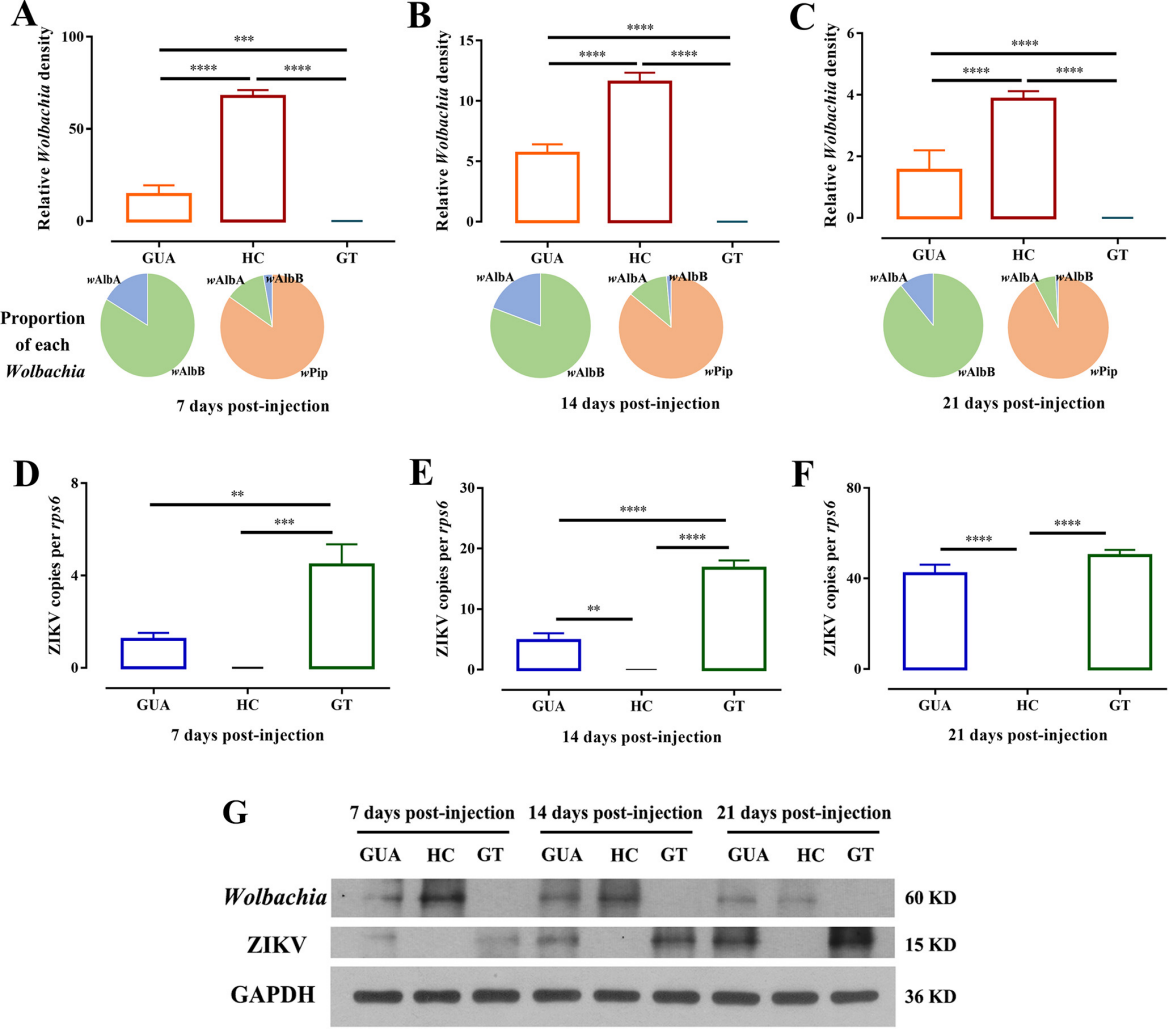
*Wolbachia* and ZIKV infection in the ovaries of *Ae. albopictus*. *Ae. albopictus* mosquitoes were infected with ZIKV by thoracic inoculation. The *Wolbachia* densities for each strain (A to C) and genome copies of ZIKV (D to F) in the ovaries of GUA, HC, and GT mosquitoes at 7, 14, and 21 days postinjection were measured by quantitative real-time PCR (qRT-PCR). Bars show average fold change per experiment ± SDs (**, *P* < 0.01; ***, *P* < 0.001; ****, *P* < 0.0001). (G) Western blots showing protein levels of *Wolbachia* heat shock protein 60 (hsp60) and ZIKV envelope at 7, 14, and 21 days postinfection in GUA, HC, and GT adult female ovaries. Twenty-five ovaries were included in one sample for analysis, and GAPDH protein was used as a control.

Thorax inoculation was chosen to positively infect mosquitoes, ensuring an even distribution of ZIKV among individual mosquito lines. The *Wolbachia* titers and the number of ZIKV in the ovaries were measured by quantitative real-time PCR (qRT-PCR) at 7, 14, and 21 days postinjection. The results showed that *Wolbachia* titers in the ovaries of HC females were significantly greater than those in GUA females, regardless of developmental stage (*P* < 0.0001, two-way ANOVA) (Fig. 3A to C). *Wolbachia* titers in ovaries of GUA and HC mosquitoes sharply decreased at 14 days postinjection and continued to decrease at 21 days postinjection (Fig. 3A to C). In GUA, *w*AlbB was the dominant strain, while in HC, *w*Pip accounted for over 85% of total *Wolbachia* density and *w*AlbB suppressed to a minimum (Fig. 3A to C). No *Wolbachia* were detected in the ovaries of GT mosquitoes (Fig. 3A to C). Conversely, the number of ZIKV genome copies in the ovaries of GUA and GT mosquitoes sharply increased at 14 days postinjection and continued to increase at 21 days postinjection (Fig. 3D to F). At 7 and 14 days postinjection, the average virus titer in the ovaries of GT mosquitoes was significantly higher than that in the ovaries of GUA mosquitoes (Fig. 3D and E). The relative density of ZIKV was similar in GUA (mean ± standard deviation [SD], 42.14 ± 3.96) and GT (45.38 ± 2.61) ovaries at 21 days postinjection (Fig. 3F). All of the HC ovarian samples were negative for ZIKV infection (Fig. 3D to F). The difference in the levels of ZIKV infection among the GUA, HC, and GT groups highlights the crucial role of *Wolbachia* in ZIKV transovarial transmission blockade.

Western blotting was conducted to quantify *Wolbachia* heat shock protein 60 (hsp60) and ZIKV envelope protein levels at 7, 14, and 21 days postinjection in adult female *Ae. albopictus* ovaries to verify qRT-PCR data. For each treatment, 12 ovaries were pooled as a sample for analysis. The blots showed higher heat shock protein 60 levels and lower ZIKV envelope protein levels in the ovaries of *Ae. albopictus* females (Fig. 3G, Fig. S4), consistent with the qRT-PCR results.

**FIG 4:**
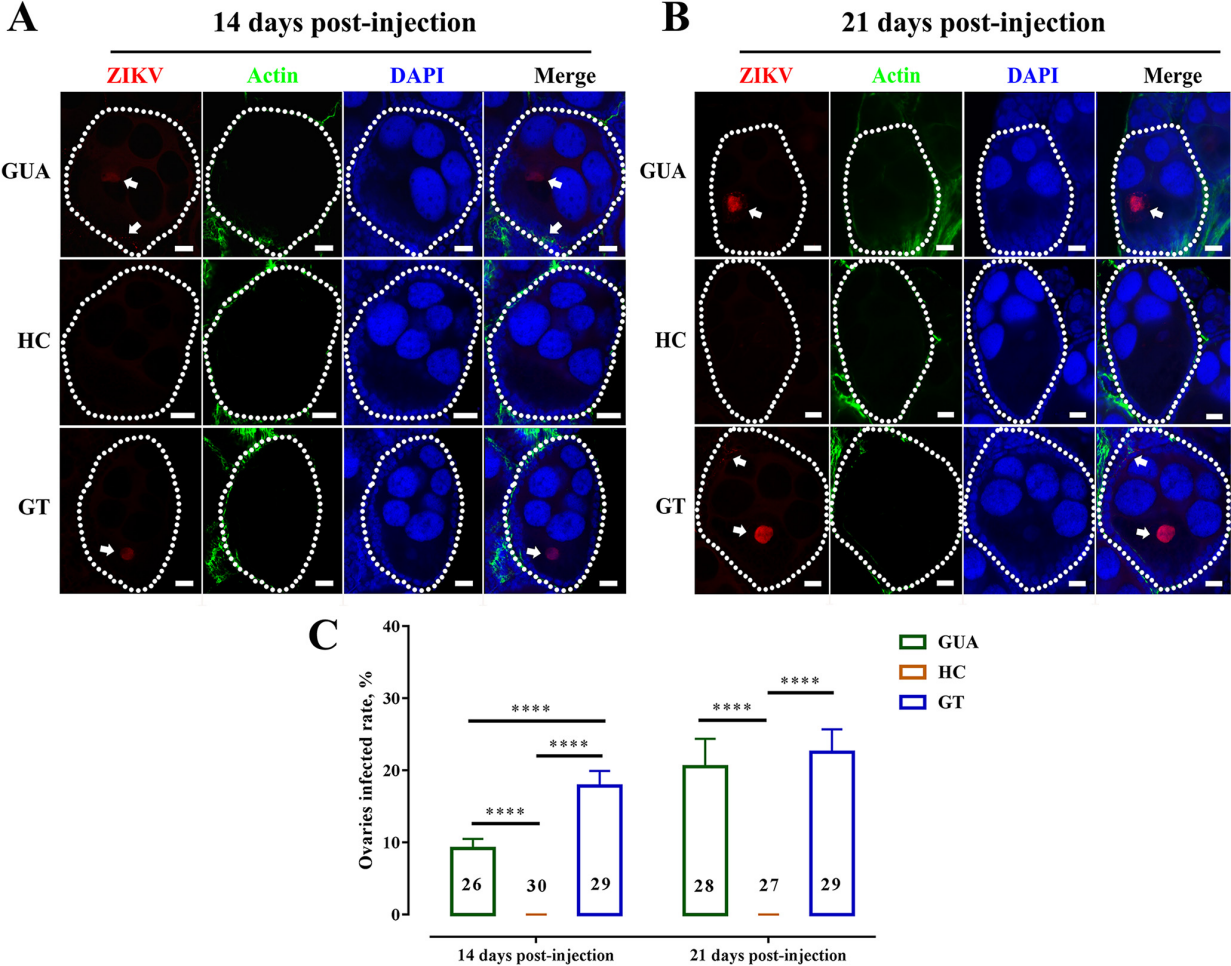
ZIKV infection at 14 and 21 days postinfection in *Ae*. *albopictus* ovaries. *Ae. albopictus* mosquitoes were infected with ZIKV by thorax inoculation. Ovaries of *Ae. albopictus* at 14 (A) and 21 (B) days postinfection were stained for ZIKV envelope (red), actin (green), and DAPI (blue) to check ZIKV infection by Z-series stack scanning. (C) ZIKV infection quantification. ZIKV envelope-labeled (red) ovaries at both 14 and 21 days postinfection were counted and expressed as a percentage of the total number of examined ovaries. Bars show the average percentages per experiment ± SDs (****, *P* < 0.0001); numbers in black show the total number of examined ovaries.

Immature ZIKV virions assemble on the surface of the host cell endoplasmic reticulum (ER), and newly formed nucleocapsids bud into the ER lumen (30). The amount of ZIKV in the ovaries of GUA and GT mosquitoes at 7 days postinjection was low. Probing with the ZIKV envelope antibody (red fluorescence) revealed extensive ZIKV around the oocyte nuclei in GUA and GT ovaries at 14 and 21 days postinjection (Fig. 4A and B, white arrowheads). No ZIKV signals were found in the ovaries of HC mosquitoes (Fig. 4A and B). We further investigated ZIKV signals using Z-stack scanning. A total of 55 ovaries from *Ae. albopictus* GUA and GT adult females were examined 14 days postinjection, of which 8 (14.5%) were observed to contain ZIKV signals (Fig. 4C). The number of ZIKV-positive ovaries in GT mosquitoes was significantly higher than that in GUA mosquitoes (Fig. 4C). The percentages were increased by 21 days postinjection in GUA and GT adults (Fig. 4C). These results indicate that *Wolbachia* infection suppresses ZIKV entry into oocytes and that the level of inhibition may depend on the host-*Wolbachia* partnership and the *Wolbachia* strain. As the number of infected oocytes and the genome copies of ZIKV in oocytes can be variable over the complex process of oogenesis, it is difficult to quantify the precise number of ZIKV-infected oocytes to determine the correlation between the number of infected oocytes and ZIKV infected progeny.

### Effect of temperature on ZIKV transovarial transmission.

**FIG 5:**
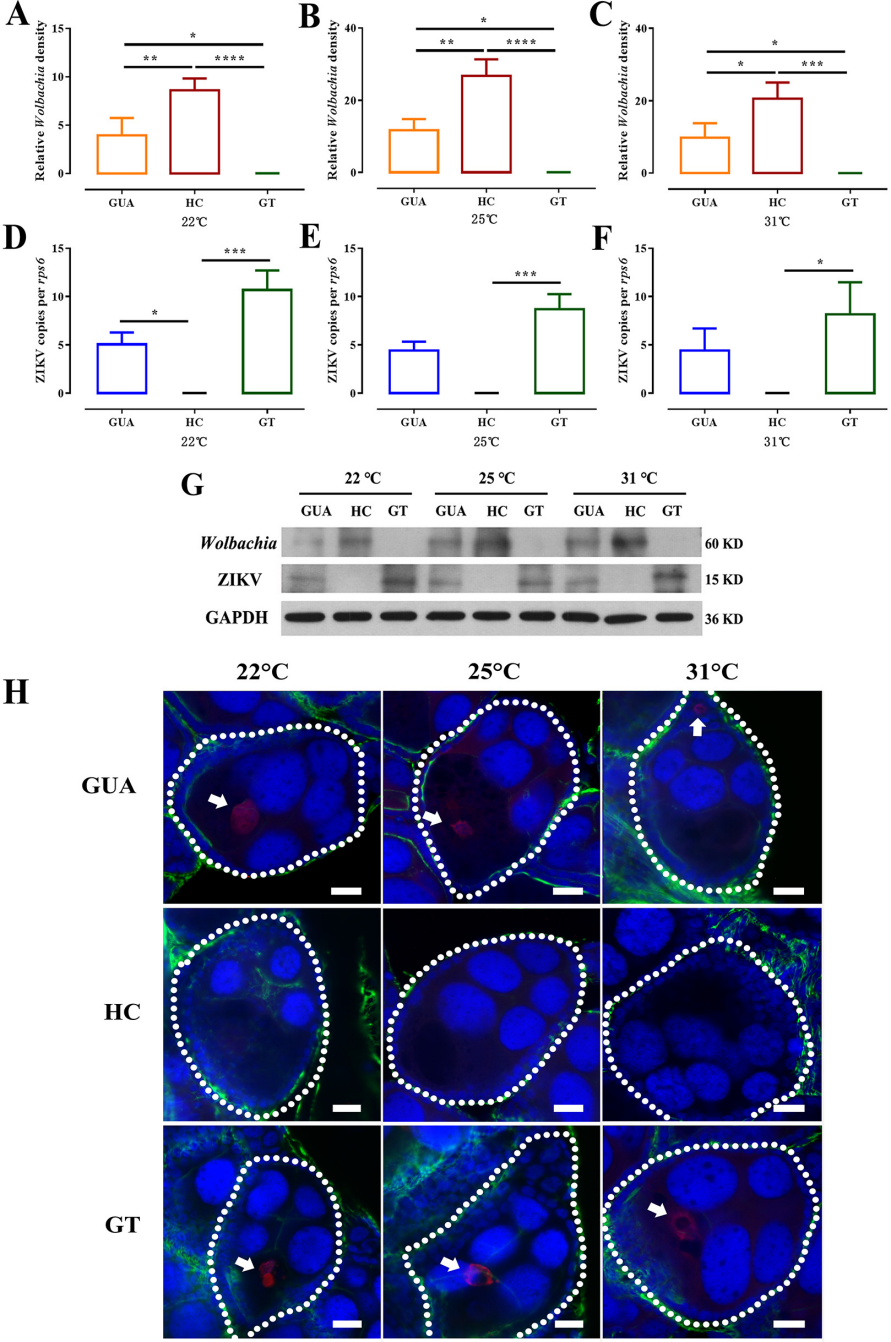
Impact of *Wolbachia* on ZIKV infection under different temperatures. The *Wolbachia* densities (A–C) and genome copies of ZIKV (D–F) in the ovaries of GUA, HC, and GT mosquitoes at 22°C, 25°C, and 31°C were measured by qRT–PCR. Bars show the average fold changes per experiment ± SDs (*, *P* < 0.05; **, *P* < 0.01; ***, *P* < 0.001; ****, *P* < 0.0001). (G) Western blotting of the protein levels of *Wolbachia* hsp60 and ZIKV envelope in the ovaries of GUA, HC, and GT adult females reared at 22°C, 25°C, and 31°C. Twenty-five ovaries were included in one sample for analysis, and GAPDH (glyceraldehyde-3-phosphate dehydrogenase) protein was used as a control. (H) Visualization of ZIKV in developing follicles by immunofluorescence staining. Red: ZIKV; green: actin; blue: *Ae. albopictus* DNA.

*Wolbachia* density is sensitive to temperature (31, 32). To investigate whether ZIKV transovarial transmission is influenced by the environment, we reared GUA, HC, and GT mosquitoes infected with ZIKV by thorax inoculation under different temperatures (22°C, 25°C, and 31°C). We then measured the correlation between ZIKV genome copy numbers and *Wolbachia* densities in the ovaries at 14 days postinjection. The results showed that the density of *Wolbachia* in the ovaries of *Ae. albopictus* was higher at 25°C than at either 22°C or 31°C (Fig. 5A to C). *Wolbachia* titers in HC ovaries were significantly greater than those in GUA female ovaries regardless of temperature (*P* < 0.05, two-tailed *t* test) (Fig. 5A to C).

The number of ZIKV genome copies as measured by qRT-PCR was significantly lower in HC ovaries than in GT ovaries, regardless of the rearing temperature (*P* < 0.05, two-tailed *t* test) (Fig. 5D to F). The ZIKV genome copy numbers in HC ovaries were significantly lower than those in GUA ovaries at 22°C, while no significant difference was found at either 25°C or 31°C (Fig. 5D to F). These results were also confirmed by Western blotting (Fig. 5G, Fig. S5) and confocal laser scanning microscopy (Fig. 5H, white arrowheads). Overall, our results suggested that the ZIKV transovarial transmission inhibition observed in HC mosquitoes was attributable to *Wolbachia*-mediated viral inhibition. The extent to which *Wolbachia* in HC and GUA mosquitoes inhibits ZIKV transovarial transmission may directly relate to environmental temperature.

### *Wolbachia* regulates immune gene expression.

In insects, innate immunity is important in limiting pathogen infection by the production of antimicrobial peptide effector molecules (33, 34). Given the reduced ZIKV genome copy numbers in HC ovaries, we reasoned that increased immune responses might partially account for decreased ZIKV proliferation. We used qRT-PCR to assess changes in the expression profiles of four effector genes (*LRIM16*, *CECE*, *DEFA*, and *DEFE*) encoding antimicrobial peptides, which were chosen based on a previous study (35, 36).

**FIG 6:**
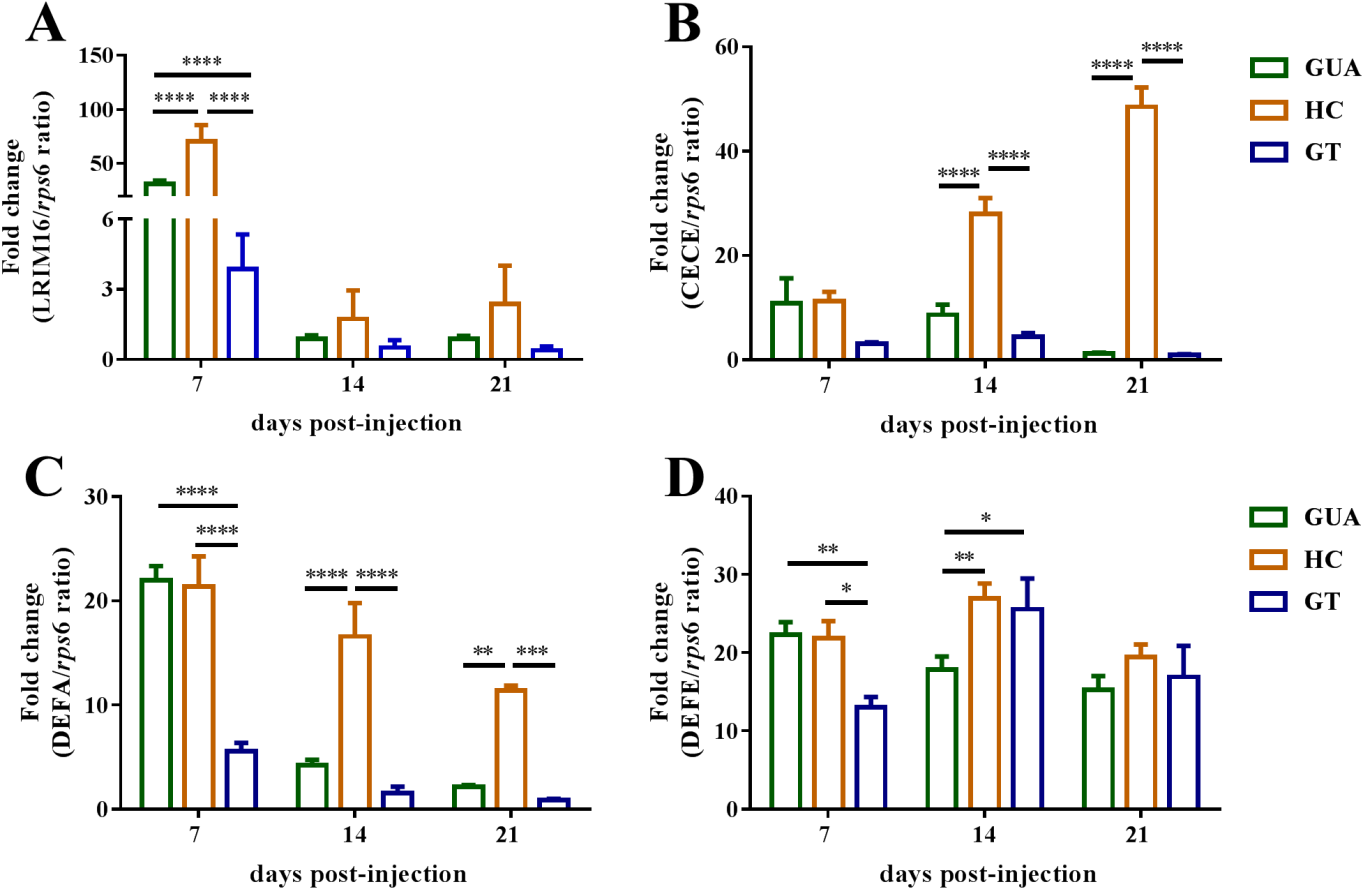
Expression of antimicrobial peptides. Transcript levels of the antimicrobial peptides LRIM16 (A), CECE (B), DEFA (C), and DEFE (D) at 7, 14, and 21 days postinfection in *Ae. albopictus* adult female ovaries were measured by qRT-PCR. Bars show average fold changes per experiment ± SDs (*, *P* < 0.05; **, *P* < 0.01; ****, *P* < 0.0001).

The expression level of the LRIM16 gene in HC ovaries was significantly higher than that in either GUA (*P* < 0.0001, two-way ANOVA) or GT (*P* < 0.0001) ovaries at 7 days postinjection (Fig. 6A). At 14 and 21 days postinjection, the expression levels of the CECE and DEFA genes were significantly higher in HC ovaries than in GT ovaries (*P* < 0.0001) (Fig. 6B and C). The LRIM16, DEFA, and DEFE genes were expressed at higher levels in GUA ovaries than in GT ovaries at 7 days postinjection (*P* < 0.01) (Fig. 6A, C, and D). Taken together, these data demonstrate that *Wolbachia* infection in HC and GUA mosquitoes strongly increased the expression of immune effector genes in the ovaries of *Ae. albopictus*.

**FIG 7:**
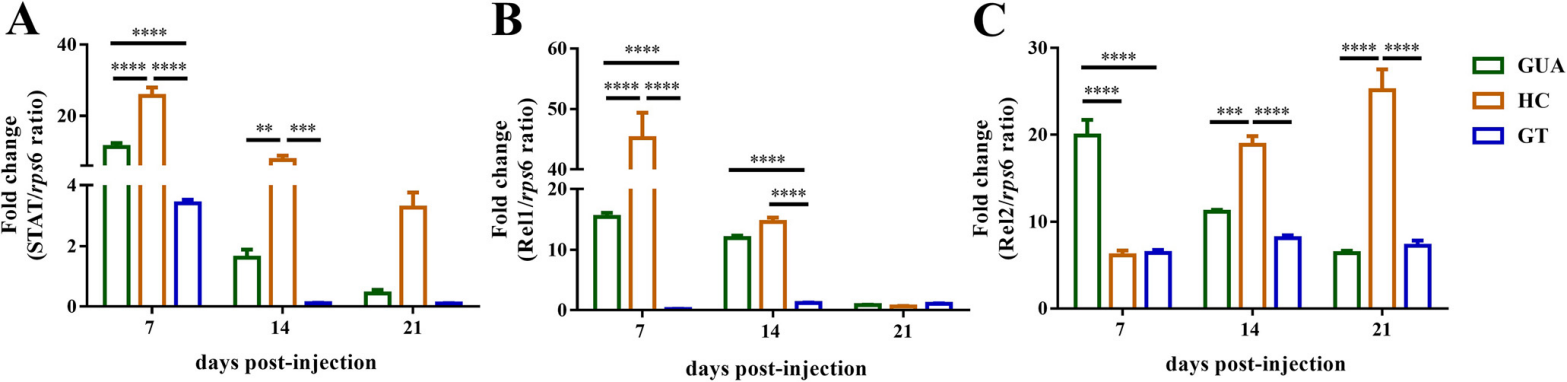
Expression of immune pathway genes. Transcription levels of the immune genes *STAT* (A), *Rel1* (B), and *Rel2* (C) at 7, 14, and 21 days postinfection in *Ae. albopictus* adult female ovaries were measured by qRT-PCR. Bars show average fold changes per experiment ± SDs (**, *P* < 0.01; ***, *P* < 0.001; ****, *P* < 0.0001).

Antimicrobial peptides are regulated by three major immune pathways: Janus kinase/signal transducer and activator of transcription (Jak/Stat), Toll, and immune deficiency (IMD) (35, 37–39). We further tested the expression levels of STAT, Rel1, and Rel2, which play central roles in the Jak/Stat, Toll, and IMD pathways, respectively (40, 41). The results showed that the expression of the STAT and Rel1 genes in HC ovaries was significantly higher than that in either GUA (*P* < 0.0001, two-way ANOVA) or GT ovaries (*P* < 0.0001) (Fig. 7A) and that the expression of *STAT*, *Rel1*, and *Rel2* was significantly higher in GUA ovaries than in GT ovaries at 7 days postinjection (*P* < 0.0001) (Fig. 7A to C). In contrast, the expression of *Rel2* was significantly higher in HC ovaries than in either GUA (*P* < 0.001) or GT ovaries (*P* < 0.0001) at 14 and 21 days postinjection (Fig. 7C).

**FIG 8:**
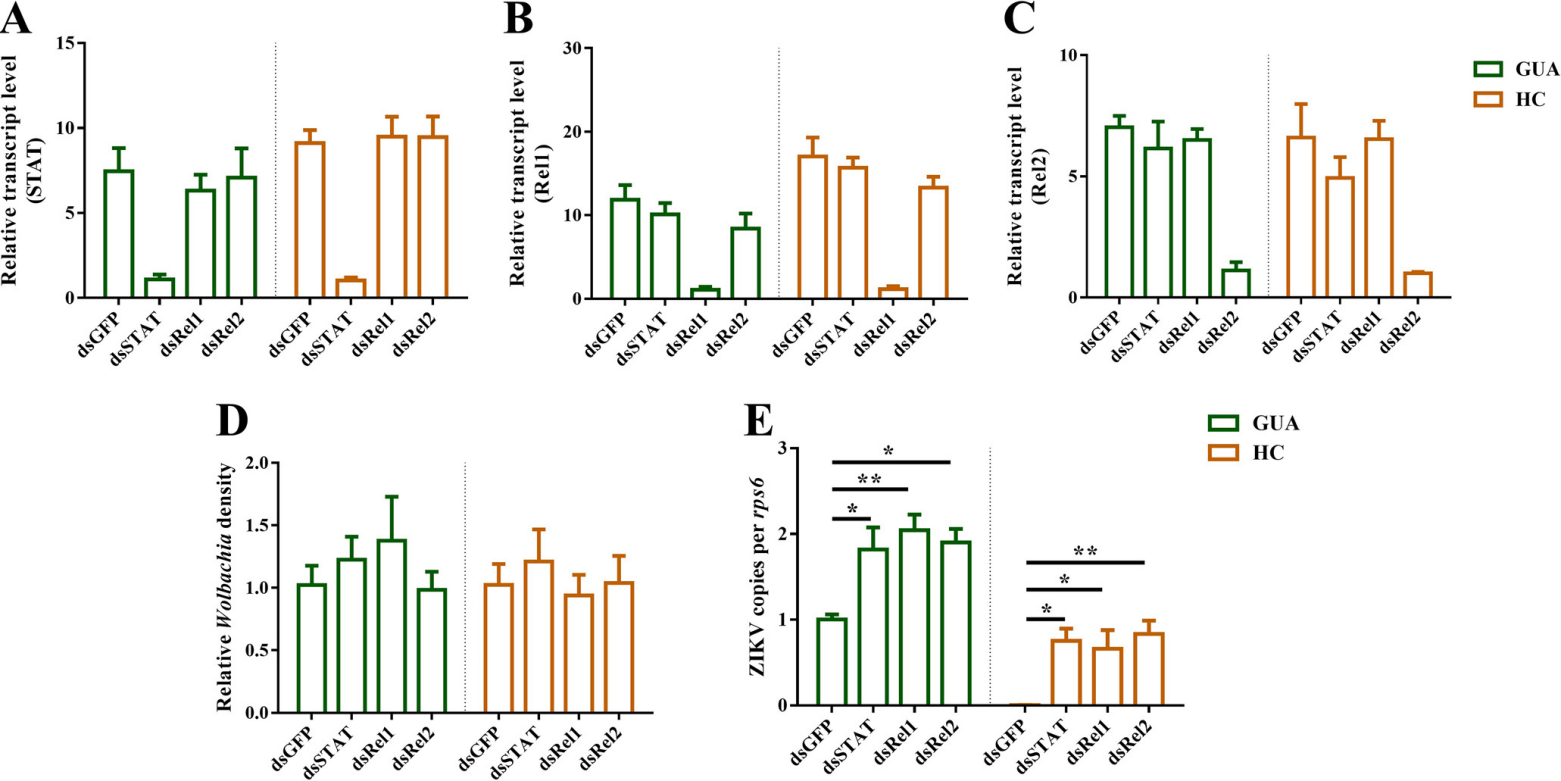
Silencing of immune gene expression in *Ae. albopictus*. Low *STAT* (A), *Rel1* (B), and *Rel2* (C) mRNA levels were detected in RNA interference (RNAi)-treated ovaries. Bars show the average relative transcription levels per experiment ± SDs. *Wolbachia* densities (D) and genome copies of ZIKV (E) in the GUA, HC, and GT RNAi-treated ovaries were measured by qRT-PCR. Bars show average fold changes per experiment ± SDs (*, *P* < 0.05; **, *P* < 0.01; ***, *P* < 0.001; ****, *P* < 0.0001).

We knocked down *STAT*, *Rel1*, and *Rel2* expression in GUA and HC females, using RNA interference (RNAi) to investigate whether immune gene knockdown in ovaries led to a loss of ZIKV inhibition. The mRNA levels of *STAT*, *Rel1*, and *Rel2* in adult ovaries and carcasses were greatly reduced (Fig. 8A to C and Fig. S6A, B, and C). There was no significant effect on *Wolbachia* titers in RNAi-microinjected ovaries and carcasses (Fig. 8D and Fig. S6D). The number of ZIKV genome copies in RNAi-microinjected female ovaries was significantly increased compared with that in dsGFP (green fluorescent protein [GFP] double-stranded RNA [dsRNA])-microinjected female ovaries (Fig. 8E). The number of ZIKV genome copies in carcasses was higher than that in dsGFP-microinjected females at the same time (Fig. S6E). Thus, there were lower ZIKV loads in the HC and GUA mosquito ovaries, which may be related to the innate immunity of mosquitoes as affected by STAT in the JAK-STAT pathway, Rel1 in the Toll pathway, and Rel2 in the IMD pathway.

## DISCUSSION

ZIKV has become a threat to public health because it has been demonstrated to cause microcephaly in neonates and has rapidly spread in many countries (42, 43). Most experiments have focused on ZIKV transmission in *Ae. albopictus* via horizontal and vertical transmission, and most studies have found that vertical transmission rates in eggs and progeny adults were low (4, 5, 44, 45). However, even at a low rate, ZIKV vertical transmission has been suggested as an important mechanism for maintaining the virus in the environment during unfavorable conditions (46). In this study, we demonstrated that GUA and GT mosquitoes could vertically transmit ZIKV to their progeny, and that the offspring of GUA and GT mosquitoes produced during the second and third gonotrophic cycles displayed higher infection rates than those produced in the first gonotrophic cycles, which is consistent with findings in previous reports (5, 6). Vertical transmission in mosquitoes occurs through trans-egg transmission and transovarial transmission, which relies on viral infection of developing oocytes. ZIKV entry into the mosquito ovary is the first step of efficient transovarial transmission. It is possible that GUA and GT ovarian infection increases during the second and third gonotrophic cycles due to the timing of dissemination of ZIKV to the ovaries and to morphological alterations of ovaries following egg formation. Stabilized infection may result in fewer females and yield high vertical transmission rates in subsequent generations. This hypothesis has also been confirmed by several studies (6, 47). Nevertheless, all examined progeny of HC mosquitoes from three gonotrophic cycles were negative for ZIKV infection. In HC mosquitoes, an almost complete blockade of ZIKV transmission was observed, suggesting that *Wolbachia* prevents ZIKV entry into the oocyte.

The different ZIKV vertical transmission rates among GUA, HC, and GT mosquitoes indicate that *Wolbachia w*Pip may inhibit virus transmission. The protective effect of *Wolbachia* against RNA viruses has been deliberately deployed for mosquito-borne disease control, and there is great interest in this area. In 2008, it was first found that *Wolbachia* protect Drosophila melanogaster against RNA viruses (48, 49). After that, *Wolbachia* were discovered to block DENV replication in mosquitoes (21, 50). Subsequently, natural infection with *Wolbachia* was found to limit virus replication and transmission in *Ae. albopictus* (51), *Aedes fluviatilis* (52), and *C. quinquefasciatus* (23), although at a lower level than in mosquito-transinfected strains (22–24). Many studies have shown that the introduction of a nonnative *Wolbachia* infection promotes an enhanced antiviral response (1, 5, 17) and that protection generally occurs in artificial host-*Wolbachia* interactions (53). Our results also point to a strong negative linear correlation between *Wolbachia* density and ZIKV load (Extended Data Table 1 in Supplemental File 1). *Wolbachia* in native infected GUA mosquitoes exhibited a negative effect on ZIKV, although slightly lower than that in HC mosquitoes. Immunofluorescence staining experiments further confirmed our results. To be transovarial transmitted, ZIKV needs to invade the host ovaries to ensure its presence in the germ line. In GUA and HC mosquitoes, *Wolbachia* was distributed among all ovarian cells, including follicular cells, the extracellular space between nurse cells, and the ooecium of oocytes. Abundant ZIKV was observed around the oocyte nuclei of GUA and GT mosquitoes, while lower numbers were located in other ovary cells. There was no obvious coexistence area for *Wolbachia* and ZIKV in the ovaries of GUA mosquitoes. As expected, high levels of *Wolbachia* and no ZIKV were observed in the ovaries of HC mosquitoes, whereas no *Wolbachia* and high levels of ZIKV were observed in the ovaries of GT mosquitoes. qRT-PCR and Western blotting also confirmed these results. High densities of *Wolbachia* induced high levels of resistance to ZIKV, and low densities exhibited limited inhibition, reflecting a possible proscriptive association between *Wolbachia* and ZIKV in GUA, HC, and GT mosquitoes. Otherwise, the degree to which *Wolbachia* interferes with ZIKV varies among GUA, HC, and GT mosquitoes, and whether it depends on *Wolbachia* density within the host or on virus identity needs further verification.

Although it is still unclear how *Wolbachia* inhibits ZIKV in the ovaries of mosquitoes, mechanisms such as competing for resources and boosting host immune responses have been proposed (21, 50, 54). Recent studies have shown that ZIKV infects mosquitoes, changing the expression of antimicrobial peptides, including LRIM16, CECE, DEFA, DEFE, and so on (35, 36). In this study, the expression level of the LRIM16 gene in HC ovaries was significantly higher than that in either GUA or GT ovaries at 7 days postinjection. At 14 and 21 days postinjection, the expression levels of the CECE and DEFA genes were significantly higher in HC ovaries than in GT ovaries. In addition, the LRIM16, DEFA, and DEFE genes were expressed at higher levels in GUA ovaries than in GT ovaries at 7-days postinjection. Overall, antimicrobial peptide expression increased in GUA and HC mosquitoes, suggesting that there is no simple relationship between the inhibition exerted by *Wolbachia* on ZIKV and the host immune response. In our study, we checked the expression levels of key genes of the Jak/Stat, Toll, and IMD immune pathways. Our results showed that the expression of the STAT and Rel1 genes was significantly higher in HC ovaries than in GUA and GT ovaries regardless of the ZIKV infection period. In addition, the expression levels of *STAT*, *Rel1*, and *Rel2* were significantly higher in GUA ovaries than in GT ovaries at 7 days postinjection. Interestingly, the expression of *Rel2* was significantly higher in HC ovaries than in GUA and GT ovaries at 14 and 21 days postinjection. We thus hypothesized that the Jak/Stat and Toll pathways initially play a central role in ZIKV infection and that the IMD immune pathway has a stable function during ZIKV infection in the presence of *Wolbachia*. Nevertheless, the role of host immune responses in *Wolbachia* interference with ZIKV in GUA, HC, and GT mosquitoes following peroral infection has not been adequately assessed. Additional studies are required to accurately determine the role of host immune responses in *Wolbachia* blockade of ZIKV expansion and maintenance in the ovaries of *Ae. albopictus*.

Overall, our study showed that *Wolbachia* is able to limit ZIKV transovarial transmission in GUA and HC mosquitoes. By affecting the expression of host immune genes, *Wolbachia* maintained high and stable levels of viral blockade in HC females, suggesting that HC mosquitoes are suitable for use in *Ae. albopictus* control programs using IIT or a combination of IIT and SIT, as described previously (7, 26).

## MATERIALS AND METHODS

### Mosquito strains.

The *Ae. albopictus* HC line infected with the *w*AlbA, *w*AlbB, and *w*Pip *Wolbachia* strains was created by transferring *w*Pip from its native mosquito host, *C. pipiens*, into the *Ae. albopictus* HOU line by embryonic microinjection (17, 28). The *Ae. albopictus* GUA line, a wild-type mosquito line superinfected with two native *Wolbachia* strains, *w*AlbA and *w*AlbB, was collected from Guangzhou by our group (55). Briefly, approximately 200 larvae collected from eight different field sites were pooled and reared to adulthood to establish the line. After that, HC females were outcrossed with males of the GUA line for 10 generations to create comparable nuclear genetic backgrounds in both mosquito lines (17). The *Wolbachia*-uninfected *Ae. albopictus* GT line was generated by antibiotic treatment of GUA insects. Tetracycline hydrochloride solution (1 mg/mL) was added to a sucrose solution which was fed to GUA adults. The treatment lasted two consecutive generations until *Wolbachia* was not detectable by diagnostic PCR. After that, the GT line was maintained on an antibiotic-free sucrose solution for 10 to 12 generations to eliminate any effects of residual antibiotic. All mosquito lines were maintained at 28°C and 70% ± 10% relative humidity, with a photoperiod of 12:12 h (light:dark) in a standard mosquito-rearing room. Adult mosquitoes were provided with a fresh sucrose solution (10%) and fed mouse blood. Mosquitoes were fed on the blood of anesthetized mice according to a protocol approved by the Ethics Committee on Laboratory Animal Care of the Zhongshan School of Medicine (approval no. 2017-041).

### Oral ZIKV inoculation.

The ZIKV strain (GenBank accession no. KY379148.1) used in this study was isolated from a patient in 2016 (56, 57). ZIKV was propagated and titrated on C6/36 cells and stored at −80°C. The ZIKV titers were measured using a plaque assay, as previously reported (36). Mosquitoes were infected with ZIKV through blood-feeding (55). Briefly, 3- to 4-day-old *Ae. albopictus* adult females were starved for 24 h and fed sheep blood (Solarbio, Beijing, China) mixed with ATP (5 × 10^−3^ M) (Sigma-Aldrich, St. Louis, MO) and containing freshly propagated ZIKV supernatant (final virus titer: 5.5 × 10^5^ PFU/mL). ZIKV-mixed blood was provided to mosquitoes by glass feeders connected to an artificial water bath circulating system (Fisher) that kept the blood at 37°C. After 40 min of feeding on the mixture, the fully engorged mosquitoes were collected. Approximately 20 blood-engorged mosquitoes of each line (HC, GUA, and GT) were checked by conventional PCR to ensure that they had ingested the infectious blood meal (data not shown). The remaining blood-engorged mosquitoes were maintained in groups of 80 in cages under standard rearing conditions, and eggs were collected using wet filter paper. After the first gonotrophic cycle, these mosquitoes were re-fed with pure sheep blood, and eggs were collected from fully engorged mosquitoes until the second and third gonotrophic cycles had finished. The filter papers with eggs were kept wet for 6 or more days to allow the eggs to mature. About 500 eggs of three gonotrophic cycles of each line were randomly selected. To remove any surface-contaminating viruses, eggs were washed twice in double-distilled water (ddH_2_O) for 5 min each time; sterilized in a 5% formalin solution for 5 min, and then washed three times for 10 min in ddH_2_O. After that, eggs were hatched for subsequent mosquito rearing. Our previous studies have demonstrated that there is no significant difference in either fecundity or fertility between the HC and GUA lines (55). Forty-eight adults from each gonotrophic cycle of the inoculated female progeny were randomly selected to test for ZIKV infection using conventional PCR. The primers are listed in Extended Data Table 2 in Supplemental File 1. The PCR products were examined by 1.2% agarose gel electrophoresis according to standard procedures. All experiments were replicated three times, and ZIKV-mixed (final virus titer: 5.5 × 10^5^ PFU/mL) blood was provided to mosquitoes each time. ZIKV-positive bands were counted, and the data are presented as the average infection rate ± SD.

### RNA extraction, reverse transcription, and PCR.

Mosquitoes which finished three gonotrophic cycles were dissected directly to check their ZIKV infection status. Total RNA was extracted from the dissected tissues (ovaries/legs/carcasses) of mosquitoes 21 days postinfection (24 mosquitoes) using TRIzol reagent (Takara Bio) according to the manufacturer’s protocol. RNA was dissolved in RNase-free water and then immediately reverse-transcribed using HiScript Q RT SuperMix for qPCR (Vazyme) after being DNase-treated. cDNA was stored at −20°C for subsequent conventional PCR analyses. The primers are listed in Extended Data Table 2. The PCR products were examined as described above. ZIKV-positive bands were counted and expressed as a percentage of the total number of examined tissues.

### Immunofluorescence staining.

*Wolbachia* in ovaries of *Ae. albopictus* were stained as previously described (58, 59). In brief, ovaries from GUA, HC, and GT adult females were dissected in phosphate-buffered saline (PBS) fixed in a 4% formaldehyde solution supplemented with 0.1% Triton X-100 for 15 min. After that, ovaries were washed twice in PBS for 5 min each time, anti-*Wolbachia* hsp60 antibody (1:200, Sigma-Aldrich) was added, and they were incubated overnight at 4°C. Next, ovaries were washed three times for 15 min in PBS with 0.1% Triton X-100 and incubated at room temperature with tetramethylrhodamine isothiocyanate (TRITC)-coupled secondary antibodies (1:250, Sigma-Aldrich) for 2 h. After two washes in PBS containing 0.2% Triton X-100 for 20 min each time, the cytoskeletons of ovaries were labeled with fluorescein isothiocyanate (FITC)-conjugated phalloidin (1 U/mL, Sigma-Aldrich) and they were mounted on glass slides with DAPI (4′,6-diamidino-2-phenylindole)-containing VECTASHIELD mounting medium (Vector Laboratories). ZIKV in the ovaries of *Ae. albopictus* was stained as described above. Ovaries were incubated with an anti-ZIKV (SPH2015) envelope primary antibody (1:200, Novus Biological) and Alexa Fluor Plus 647-labeled goat anti-rabbit IgG secondary antibody (1:500, Invitrogen).

Ovaries were examined using a Zeiss LSM-800 confocal laser scanning microscope and scanned to acquire Z-series stacks at 2.0-mm intervals. The exposure times were normalized across all experiments.

### Transmission electron microscopy.

Ovaries from GUA, HC, and GT adult females were dissected, washed in PBS, and fixed in 2.5% (vol/vol) glutaraldehyde in PBS at 4°C overnight. After that, the ovaries were washed three times with 0.1 M PBS (pH 7.0) and then postfixed in 1% (vol/vol) osmium tetroxide at room temperature for 1 h. The fixed ovaries were dehydrated for 10 min each in a 50, 70, 80, 90, 95, and 100% (vol/vol) graded series of ethanol and soaked in acetone for 20 min; this was followed by placement in a 1:1 mixture of acetone and Spurr resin for 1 h, then in a 1:3 mixture for 3 h, and finally in Spurr resin alone overnight at room temperature. Next, the ovaries were polymerized for 16 h at 70°C. Glass knives on an LKB Bromma 11800 pyramitome (LKB, Bromma, Sweden) was used to cut semithin sections of ovaries, and ultrathin sections of ovaries were cut with a diamond knife using a PowerTome-PC (RMC, Boeckeler Instruments, Tucson, AZ). After staining with 3% uranyl acetate and alkaline lead citrate, ovaries were observed using TEM with a JEM-1230 model (JEOL, Tokyo Japan) at an accelerating voltage of 80 kV.

### Intrathoracic ZIKV inoculation.

To ensure an even distribution of ZIKV among individual mosquito groups, thorax inoculation was chosen to positively infect mosquitoes. ZIKV culture supernatant (5 × 10^5^ PFU/mL) was injected into female mosquitoes by thorax inoculation using a Nanoject II microinjector (Drummond). One- to two-day-old GUA, HC, and GT female mosquitoes were infected with ZIKV by intrathoracic inoculation. Each mosquito was injected with 69 nL of ZIKV supernatant and blood-fed at 2, 10, and 17 days postinjection. To verify the effect of *Wolbachia* on *Ae. albopictus* females, mosquitoes were reared at different temperatures (22°C, 25°C, and 31°C) after ZIKV injection. The number of ZIKV virus copies and *Wolbachia* densities in the ovaries of *Ae. albopictus* reared under different temperatures were measured and compared with those in the ovaries of *Ae. albopictus* reared at 28°C.

### ZIKV and *Wolbachia* quantification.

Ovaries from GUA, HC, and GT adult females at different developmental stages were dissected and collected. Total RNA was extracted from half of the ovaries, using RNAiso (Takara Bio) according to the manufacturer’s protocol. Extracted RNA was dissolved using RNase-free water and then DNase-treated and reverse-transcribed immediately using HiScript Q RT SuperMix for qPCR (Vazyme). cDNA was stored at −20°C for subsequent qRT-PCR analysis. Total DNA was extracted from the other half of the ovary sample using the phenol-chloroform method to measure *Wolbachia* genome copy numbers, dissolved in ddH_2_O, and stored at −20°C for subsequent PCR analyses.

The genome copies of ZIKV and *Wolbachia* in the ovaries from mosquitoes in different developmental stages (7, 14, and 21 days postinjection) were measured using qRT-PCR, as previously described (17, 55, 59). Before the experiment, PCR products of the ZIKV *NS1*, *Wolbachia wsp* and host *rps6* reference genes were amplified by conventional PCR, purified using an AxyPrep DNA Gel Extraction kit (Axygen), and cloned into a pEASY-T1 vector (TransGene Biotech, Beijing, China). Plasmid DNAs containing target gene fragments were used for serial dilutions of 10^−1^ to 10^−8^ to generate standard curves. Specific primers for *w*AlbA, *w*AlbB, and *w*Pip were used in qRT-PCR to individually quantify each *Wolbachia* strain. qRT-PCR was performed on a Roche LightCycler 96 machine using SYBR Premix Ex Taq (Takara Bio). The following procedures were used: denaturation at 95°C for 30 s and 40 amplification cycles (95°C for 5 s and 60°C for 30 s), followed by melting curve analysis. Primers are shown in Extended Data Table 2. Each sample was tested three times, and negative controls were included in all amplification reactions. The sample was considered negative if the quantification cycle (*Cq*) value was 35 or higher. ZIKV and *Wolbachia* copies in mosquito tissues were normalized using the mosquito *rps6* gene.

### Western blotting.

Ovaries were quickly dissected from *Ae. albopictus* adult females at 7, 14, and 21 days postinjection and then homogenized in radioimmunoprecipitation assay lysis buffer. Protein concentrations were measured using a Pierce BCA Protein assay kit (Thermo Fisher Scientific, Waltham, MA) according to the manufacturer’s protocol. After the addition of 6× SDS loading buffer, the lysates were boiled for 10 min. The proteins of *Ae. albopictus* ovaries were separated by electrophoresis on a 12% SDS-PAGE gel running at 80 to 130 V for 2 h and then transferred to a polyvinylidene difluoride membrane. After this, the membrane was probed with primary antibodies (1:5,000) and tested using horseradish peroxidase-conjugated goat anti-mouse IgG antibodies (1:10,000, Jackson ImmunoResearch, West Grove, PA). In the experiment, anti-GAPDH (glyceraldehyde-3-phosphate dehydrogenase) polyclonal rabbit serum (1:10,000, Aksomics, Shanghai, China) was used to monitor equal protein loading. Nitrotetrazolium blue chloride/5-bromo-4-chloro-3-indolyl phosphate (NBT/BCIP) buffer (Sigma-Aldrich) was used to visualize target fragments under room temperature conditions. Anti-ZIKV (SPH2015) envelope antibody (Novus Biological) and anti-*Wolbachia* hsp60 antibody (Sigma-Aldrich) were used in this study.

### Antimicrobial peptide and immune-related genes.

Insect innate immunity plays an important role in limiting pathogen infection. Activation of innate immune pathways leads to the nuclear translocation of transcription factors, resulting in the production of a variety of antipathogen effector molecules, such as antimicrobial peptides (33, 38). The expression of a number of antimicrobial peptides, including LRIM16, CECE, DEFA, and DEFE, was upregulated by ZIKV infection in *Ae. albopictus* (33, 34). We examined the timing of expression of LRIM16, CECE, DEFA, and DEFE in the ovaries of each *Ae. albopictus* line reared at 28°C by qRT-PCR at 7, 14, and 21 days postinjection, as described above.

Immune responses are regulated by the Toll, IMD, and Jak/Stat signaling pathways (38, 60, 61). STAT, Rel1, and Rel2 play central roles in the Jak/Stat, Toll, and IMD immune-signaling pathways, respectively. The timing of STAT, Rel1, and Rel2 expression in the ovaries of each *Ae. albopictus* line at 7, 14, and 21 days postinjection was measured using qRT-PCR, as described above.

### dsRNA gene silencing.

PCR-amplified gene fragments were used to synthesized double-stranded RNA using a T7 High Yield RNA Transcription kit (Vazyme). The primers are listed in Extended Data Table 3 (Supplemental File 1). dsRNA (250 ng) was injected into the thorax of 2-day-old female mosquitoes using a Nanoject II microinjector (Drummond). Assays were repeated three times, using GFP dsRNA as a control. Gene silencing efficiency was calculated by comparing the relative mRNA levels of the target gene after knockdown with its specific dsRNA and control dsRNA using qRT-PCR. Three days after injection, the mosquitoes were fed a ZIKV-supplemented blood meal. Dissection of mosquito ovaries and carcasses was performed at 7 days postinfection. The number of ZIKV virus copies and *Wolbachia* densities in the ovaries/carcasses of *Ae. albopictus* were measured using qRT-PCR.

### Statistical analysis.

SPSS version 20.0 (IBM, Chicago, IL) was used for statistical analyses. For comparisons of the genome copies of ZIKV and *Wolbachia* in the ovaries of mosquitoes, gene expression levels, and knockdown efficiency, differences among multiple samples and between two samples were compared using two-way analysis of variance (ANOVA) and Student’s *t* test, followed by Benjamini-Hochberg correction. Mann-Whitney U tests were used in a few cases when data were not normally distributed. Spearman’s correlation tests were used to investigate relationships between ZIKV and *Wolbachia* in the ovaries of mosquitoes. Differences were regarded as statistically nonsignificant at *P* > 0.05.
