# Supplementary material for: Wolbachia wPip Blocks Zika Virus Transovarial Transmission in Aedes albopictus
Source: Microbiol Spectr. 2022 Jul 27;10(5):e02633-21. doi: 10.1128/spectrum.02633-21 (PMC9603370; doi:10.1128/spectrum.02633-21)
Supplement: Supplemental file 1 — Fig. S1 to S6 and Tables S1 to S3. Download spectrum.02633-21-s0001.pdf, PDF file, 0.6 MB [file spectrum.02633-21-s0001.pdf]

**Supplementary material**

# *Wolbachia w*Pip block Zika virus transovarial transmission in *Aedes albopictus*

Yan Guo,[a*] Jiatian Guo,[b] Yifeng Li,[a] Xiaoying Zheng,[b] Yu Wu[b]

[a]Guangdong Provincial Key Laboratory of High Technology for Plant Protection, Plant Protection Research Institute, Guangdong Academy of Agricultural Science, Guangzhou, Guangdong, China

[b]Key Laboratory of Tropical Disease Control of the Ministry of Education, Zhongshan School of Medicine, Sun Yat-Sen University, Guangzhou, Guangdong, China

[*] Correspondence:

Dr. Yan Guo, Guangdong Provincial Key Laboratory of High Technology for Plant Protection, Plant Protection Research Institute, Guangdong academy of Agricultural Science, Guangzhou, Guangdong, China, yanguo20916129@163.com

## Figure legends

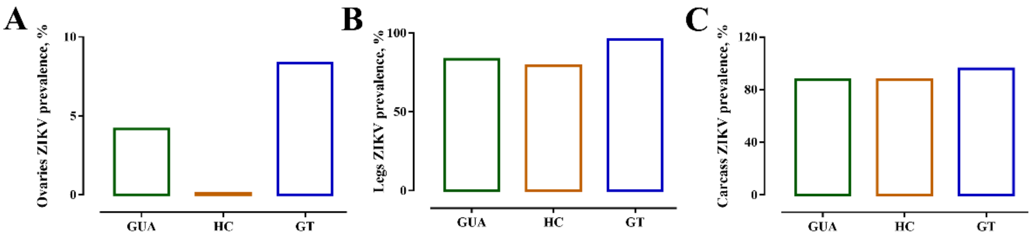


**Figure S1.** ZIKV infection in *Ae. albopictus* which finished three gonotrophic cycles. A total of 24 female mosquitos of GUA, HC and GT lines which have finished three gonotrophic cycles were dissected (ovaries/legs/carcasses) and checked their ZIKV infection status by PCR. Bars show the ZIKV infection percentages per experiment.

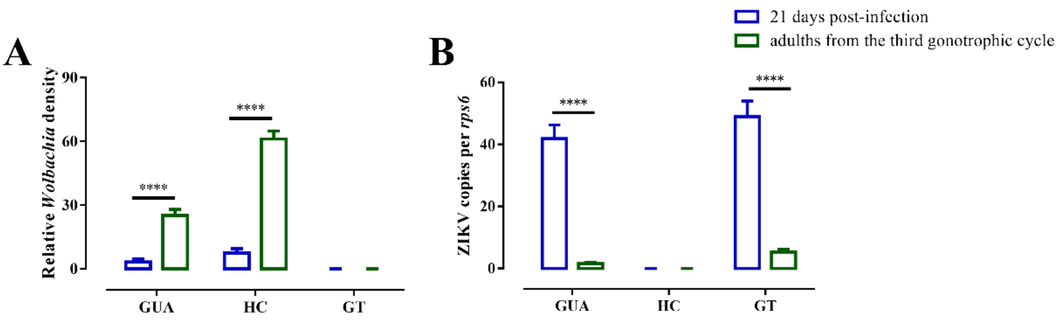


**Figure S2.** The *Wolbachia* densities (A) and genome copies of ZIKV (B) of GUA, HC and GT lines that had finished three gonotrophic cycles and adults of the third gonotrophic cycle progeny were measured by qRT-PCR. Bars show the average fold changes per experiment ± SDs (**** $P <$ 0.0001).

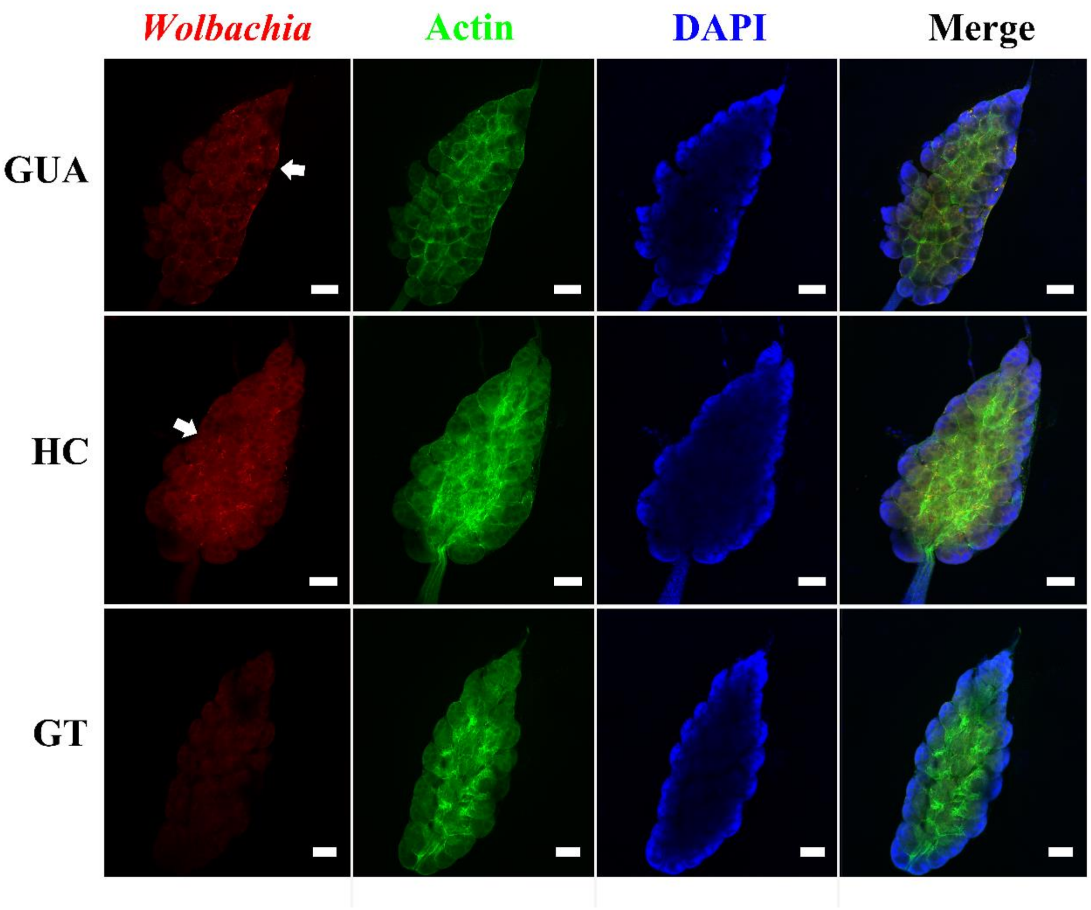


**Figure S3.** *Wolbachia* distribution in *Ae. albopictus* ovaries. *Wolbachia* distribute among all cells of ovaries. Red: *Wolbachia*; green: actin; blue: *Ae. albopictus* DNA. Scale bars: 50 μm.

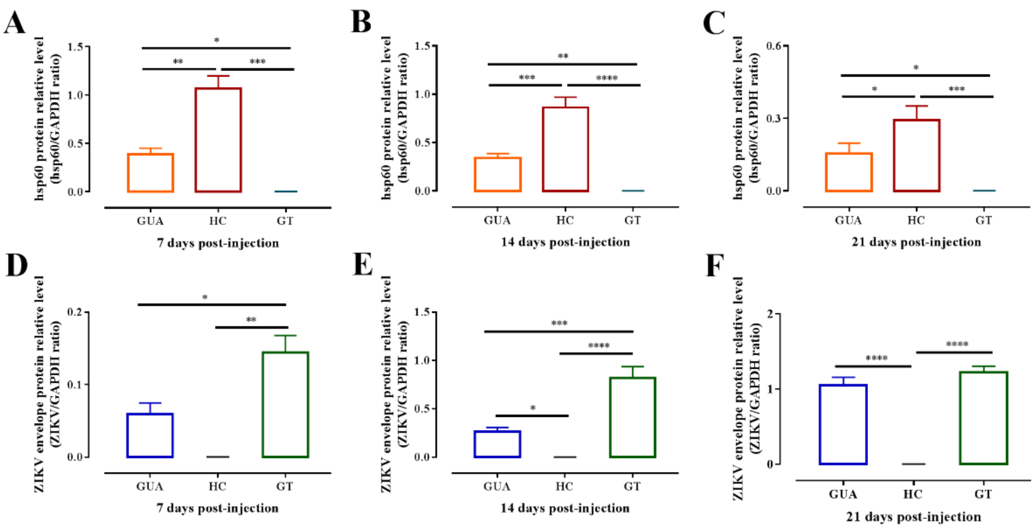


**Figure S4.** Densitometric analysis the levels of *Wolbachia* hsp60 and ZIKV envelope proteins in the ovaries of *Ae. albopictus* infected ZIKV by thorax inoculation. Bars show the average fold

changes per experiment ± SDs (* $P < 0.05$, ** $P < 0.01$, *** $P < 0.001$, **** $P < 0.0001$).

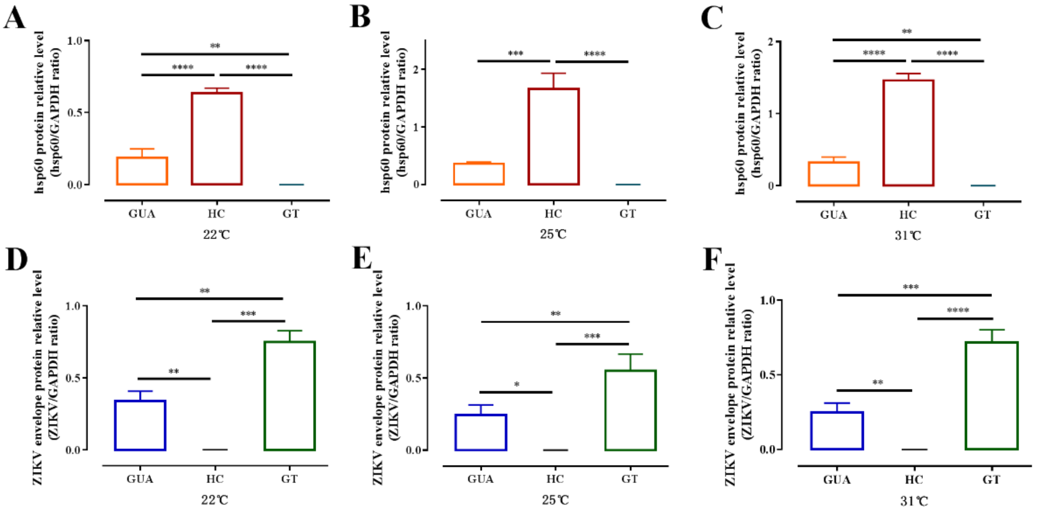


**Figure S5.** Densitometric analysis the levels of *Wolbachia* hsp60 and ZIKV envelope proteins in the ovaries of *Ae. albopictus* reared at 22°C, 25°C, and 31°C. Bars show the average fold changes per experiment ± SDs (* $P < 0.05$, ** $P < 0.01$, *** $P < 0.001$, **** $P < 0.0001$).

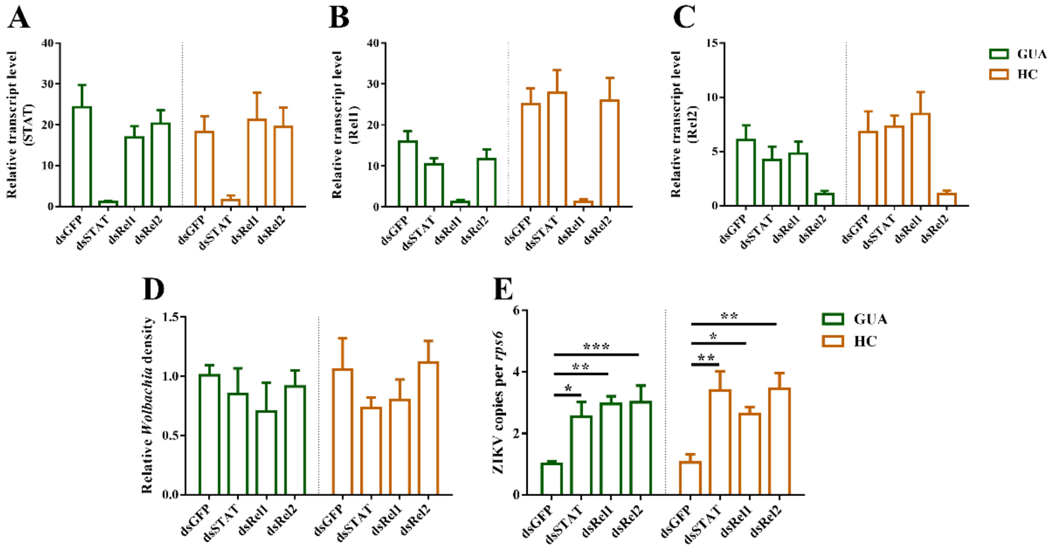


**Figure S6.** Silencing of the expression of immune genes in *Ae. albopictus*. Low STAT (A), Rel1 (B), and Rel2 (C) mRNA levels were detected in RNAi-treated carcasses. Bars show the average relative transcript levels per experiment ± SDs. The *Wolbachia* densities (D) and genome copies of ZIKV (E) in the GUA, HC, and GT RNAi-treated carcasses were measured by qRT–PCR. Bars show the average fold changes per experiment ± SDs (* $P < 0.05$, ** $P < 0.01$, *** $P < 0.001$, **** $P < 0.0001$).

**Extended Data Table 1.** Investigate relationships between ZIKV and *Wolbachia* in the ovaries of mosquitoes used Spearman's correlation tests.

| Factors | *P*-value | rho |
|---|---|---|
| 7 days post-injection | 0.001394 | -0.6261074 |
| 14 days post-injection | 1.385e-09 | -0.9120607 |
| 21 days post-injection | 6.724e-06 | -0.7919182 |
| 22°C | 6.108e-07 | -0.8592033 |
| 25°C | 2.112e-08 | -0.9030234 |
| 31°C | 8.624e-05 | -0.7785435 |

**Extended Data Table 2.** Primers used in quantitative real-time PCR and PCR.

| **Genes** | **Forward primer (5′-3′)** | **Reverse primer (5′-3′)** |
|---|---|---|
| ZIKV *NS1* | ACCCAAGTCTTTAGCTGGGC | CTGGTTCTTTCCTGGGCCTT |
| *Wolbachia wsp* | CTGGTGTTAGTTATGATGTAAC | AAAAATTAAACGCTACTCCA |
| *rps 6* | CGTCGTCAGGAACGTATTCG | TCTTGGCAGCCTTGACAGC |
| *w*AlbA | GGGTTGATGTTGAAG GAG | CACCAGCTTTTACTTGACC |
| *w*AlbB | ACGTTGGTGGTGCAACATTTG | TAACGAGCACCAGCATAAAGC |
| *w*Pip | TATTTCCCACTATATCCCTTC | GGATTTGACCTTTCCGGC |
| LRIM16 | AGGAAACCGGCTCAAGGATG | GGATTTGTTCGCGATGGACG |
| CECE | CAGCGAACCGTAAGTCACCT | TTCTTCAGACCACCGGCTTC |
| DEFA | GTGTCGTTTGCTTGGTTGCT | CCCAACACCGAATCCACTCA |
| DEFE | CATTGGTTTCCCTCTACGCG | ATTCCAGCCAAGTCCACTCA |
| Rel1 | TGGTGGTGGTGTCCTGCGTAA C | CTGCCTGGCGTGACCGTATCC |
| Rel2 | GCTCAGTGCTACCGTGGGAAA C | CGGGTTCGCTCTGGCATTTGTC |
| STAT | GCCAAAACCTGTTCCTCTTG | CGATGTAGCATTCGGTGATG |

**Extended Data Table 3.** Primers used in double stranded RNA (dsRNA) synthesis.

| **Genes** | **Sequence (5′-3′)** |
|---|---|
| dsRel1-sense | TAATACGACTCACTATAGG AGCATCGGCGAGATCAACAT |
| dsRel1-antisense | TAATACGACTCACTATAGG TCGAAACTGTCCGAGAGCGT |

| | |
|---|---|
| dsRel2-sense | TAATACGACTCACTATAGG AGTTTGAACGTTCTGCTGG |
| dsRel2-antisense | TAATACGACTCACTATAGG TTGTGTCCATGCTTCAGATC |
| dsSTAT -sense | TAATACGACTCACTATAGG CACCGGATCGTTAACCCTG |
| dsSTAT -antisense | TAATACGACTCACTATAGG AGCCATGGACACGTCGTC |
| dsGFP-sense | TAATACGACTCACTATAGGGAGAATGAGTAAAGGAGAAGAAC TTTTC |
| dsGFP-antisense | TAATACGACTCACTATAGGGAGATTTGTATAGTTCATCCATGCC ATGT |
